# Assessing health-related resiliency in HIV+ Latin women: Preliminary psychometric findings

**DOI:** 10.1371/journal.pone.0181253

**Published:** 2017-07-19

**Authors:** Gladys J. Jimenez-Torres, Valerie Wojna, Ernesto Rosario, Rosa Hechevarría, Ada M. Alemán-Batista, Miriam Ríos Matos, Alok Madan, Richard L. Skolasky, Summer F. Acevedo

**Affiliations:** 1 The Menninger Clinic, Houston, TX, United States of America; 2 Psychiatry & Behavioral Sciences, Baylor College of Medicine, Houston, TX, United States of America; 3 Psychology Department, Ponce School of Medicine and Health Sciences, Ponce, PR, United States of America; 4 NeuroAIDS Research Program, University of Puerto Rico, San Juan, PR, United States of America; 5 Department of Internal Medicine, Neurology Division, University of Puerto Rico, San Juan, PR, United States of America; 6 Physical Medicine and Rehabilitation, University of Puerto Rico, San Juan, PR, United States of America; 7 School of Health Professions, University of Puerto Rico, San Juan, PR, United States of America; 8 Department of Orthopedic Surgery and Physical Medicine & Rehabilitation, Johns Hopkins University, Baltimore, MD, United States of America; 9 Department of Psychiatry, UT Southwestern Medical Center, Dallas, TX, United States of America; Technion Israel Institute of Technology, ISRAEL

## Abstract

**Background:**

HIV-associated vulnerabilities—especially those linked to psychological issues—and limited mental health–treatment resources have the potential to adversely affect the health statuses of individuals. The concept of resilience has been introduced in the literature to shift the emphasis from vulnerability to protective factors. Resilience, however, is an evolving construct and is measured in various ways, though rarely among underserved, minority populations. Herein, we present the preliminary psychometric properties of a sample of HIV-seropositive Puerto Rican women, measured using a newly developed health-related resilience scale.

**Methods and design:**

The Resilience Scales for Children and Adolescents, an instrument with solid test construction properties, acted as a model in the development (in both English and Spanish) of the HRRS, providing the same dimensions and most of the same subscales. The present sample was nested within the Hispanic-Latino longitudinal cohort of women (HLLC), that is part of the NeuroAIDS Research Program at the University of Puerto Rico (UPR), Medical Sciences Campus (MSC). Forty-five consecutively recruited, HIV+ women from the HLLC completed a demographic survey, the HRRS, and the Beck Depression Inventory-I, Spanish version.

**Results:**

The results demonstrate excellent overall internal consistency for the total HRRS score (α = 0.95). Each of the dimensional scores also evidenced acceptable internal consistency (α ≥ 0.88). All the dimensional and subscale content validity indices were above the 0.42 cut-off. Analysis revealed a significant negative correlation between the HRRS total score and BDI-I-S (r(45) = -0.453, *p* < 0.003).

**Conclusion:**

Albeit preliminary in nature, the present study provides support for the HRRS as a measure to assess resilience among individuals living with chronic medical conditions. Minority populations, especially non-English speaking ones, are understudied across the field of medicine, and when efforts are made to include these patient groups, measurement is rarely tailored to their unique cultural and linguistic experiences. The HRRS is a measure that addresses these notable voids in the medical literature.

## Introduction

In 2010, there were 10,731 new cases of HIV/AIDS in Hispanics; 15.8% of those cases were in Puerto Rico, alone [1]. The rates of women contracting the disease were higher in Puerto Rico (24.7%) than in the continental US (15.95%) [1]. HIV+ Puerto Rican women are at increased risk for developing depressive symptoms [2, 3], neurocognitive impairments associated with HIV [4–6], having poor adherence to therapy [7] and frequently experiencing violence [8],. Limited available resources constrain public health efforts in Puerto Rico to provide care for people living with HIV/AIDS [9], including hindering those efforts aimed at implementing evidence-based mental health interventions [10].

HIV-associated vulnerabilities—especially those linked to psychological issues—and limited mental health–treatment resources have the potential to adversely affect the health status of HIV+ Puerto Rican women by increasing stress [11–13]. A number of factors can affect the extent to which an individual feels capable of managing these vulnerabilities, in turn influencing his or her ability to cope with stressors. Cognitive appraisal enables individuals to exercise their judgment and assess the significance of stress (primary appraisal) and the coping options available within that individual’s means (secondary appraisal). In this study, we address primary cognitive appraisal as a mechanism underlying HIV+ Puerto Rican women’s resilience to stressors.

The concept of resilience has been introduced in the HIV/AIDS literature [14–18] to shift the emphasis from vulnerability to protective factors, especially taking into consideration flexibility as a means of facilitating healthy adaptation and potentially decreasing vulnerability to psychiatric disorders [19]. Resilience has been identified as a protective factor for individuals living with HIV/AIDS, from developing clinically significant depression [18, 20, 21]. Supportive relationships, emotion regulation, and sense of mastery have been identified as key dimensions that foster resilience [22, 23]. Resilience, however, is an evolving construct that is operationalized and measured in various ways [24].

Herein, we introduce the term *health-related resilience* (HRR), defined as an individual’s ability to make inferences regarding a given health-related stressor or adverse event and to withstand the difficulties associated with the stressor or, alternately, to overcome the stress caused by the adverse event. [25]. Resilience not only relates to an individual’s cognitive–behavioral capabilities but also to that individual’s ability to properly identify and use available resources to help him or her cope with the health-related stressors by modifying that stressor, thereby adapting to the required changes [24, 26]. Resilience provides an individual with a way to identify both adaptive and maladaptive approaches in the regulation of stress, potentially affecting that person’s ability to benefit from mental health treatment [27, 28]. Despite the growing recognition of the influence of resilience in influencing a person’s ability to cope with the stressors of living with HIV, few studies have assessed resilience in HIV+ adults [29]; fewer have assessed resilience in Hispanic HIV+ adults [30–32], especially Hispanic HIV+ women [2, 18, 33]. The complex socio-demographic characteristics of this underserved population are notably missing from the HIV literature.

Despite a growing interest in studying resilience, little empirical research has addressed the associated multidimensional nature of the construct. A theory-based instrument measuring constructs previously associated with resilience exists, but it was intended for use with children and adolescents [34]. The Dispositional Resilience Scale (DRS) [35] and the Connor-Davidson Resilience Scales (CS-RS) [36, 37] have been used to evaluate HIV+ individuals [18, 38–41]. However, the DRS was originally intended to measure the impact of an air disaster on health [35], and the CD-RS, though originally developed in various cohorts of adults, has been used in children and adolescents to measure childhood trauma [36, 37]. Other well-known scales have examined resilience to developmental changes [42] and resilience as a personality trait [43–45]. To date, most scales and interventions have focused on risk factors, not being intended specifically as tools to be used in the development of recommendations for patients who live with comorbid mental health and chronic physical conditions, such as do many HIV+ individuals. To fill this notable void, we developed the Health-Related Resilience Scale (HRRS) and present herein the preliminary psychometric properties from a pilot sample of HIV+ Puerto Rican women.

## Methods

### Scale development

The Resiliency Scales for Children and Adolescents, an instrument with solid test construction properties, acted as a model in the development (in both English and Spanish) of the HRRS, providing the same dimensions and most of the same subscales. The *sense of mastery* dimension (SMD) is composed of the optimism, self-efficacy, and adaptation subscales. The *sense of relatedness* dimension (SRD) is composed of the trust, social support, comfort, and tolerance for differences subscales. The *emotional reactivity* dimension (ERD) is composed of the sensitivity, recovery, and balance subscales. The balance subscale is not in the RSCA, which uses instead a subscale for impairment [34]; the substitution was made in the HRRS in the interest of emphasizing strengths rather than weaknesses. The construction of the HRRS followed DeVillis’s (2003) guidelines for scale development [46]. Although the HRRS applied most of the concepts for dimensions and subscales that are present in the RSCA, the HRRS’s operational definitions for dimensions and subscales, as well as for individual items, were created using an inductive approach [47]. Each item is rated on a 5-point, Likert-type scale, ranging from *never* (0) to *always* (4) (See Table 1). Items and instructions for respondents were initially created in the Spanish language and were written at the sixth-grade reading level. To confirm the consistency of its content, the scale was translated from the Spanish to the English language, and then back-translated from the English to the Spanish language by two different qualified bilingual clinical psychologists. See Table 2 for the Spanish version of the HRRS.

**Table 1:** Health-Related Resiliency Scale in English. INSTRUCTIONS: Read each phrase carefully. Circle the answer that best indicates the way you thought and felt (never, rarely, sometimes, frequently, always) about each one in the previous 7 days. Remember that there are no right or wrong answers.

Dimensions	Never	Rarely	Sometimes	Frequently	Always
**Sense of Mastery Dimension -**					
I visualize my future with hope.	0	1	2	3	4
I try to see the good side of things.	0	1	2	3	4
I can identify the benefits that result from my situation.	0	1	2	3	4
I trust my capacity to make decisions.	0	1	2	3	4
I develop effective solutions to my problems.	0	1	2	3	4
I derive satisfaction from my achievements	0	1	2	3	4
I can achieve what I want under stress.	0	1	2	3	4
I ask for help when I need it.	0	1	2	3	4
I can adjust to whatever life brings.	0	1	2	3	4
**Emotional Reactivity Dimension -**					
I stay calm even when I am stressed.	0	1	2	3	4
I manage my emotional commitments positively even when I am stressed.	0	1	2	3	4
I maintain serenity during difficult situations.	0	1	2	3	4
I recover from stressful situations in a short time.	0	1	2	3	4
I manage to regain tranquility after facing difficulties.	0	1	2	3	4
I deal effectively with my emotions during difficult situations.	0	1	2	3	4
Even when facing difficulties, I maintain tranquility.	0	1	2	3	4
I keep my emotions in control most of the time.	0	1	2	3	4
I regulate my emotions according to the situations I face.	0	1	2	3	4
**Sense of Relatedness Dimension**					
I trust other people.	0	1	2	3	4
I have let others know how I feel.	0	1	2	3	4
I can delegate to others.	0	1	2	3	4
Other people understand the things I worry about.	0	1	2	3	4
I go to others for advice or guidance.	0	1	2	3	4
There are people who love and care for me.	0	1	2	3	4
I maintain lasting relationships.	0	1	2	3	4
My relationships with other people help me see things from another point of view.	0	1	2	3	4
I feel comfortable in my relationships.	0	1	2	3	4
I respect differences in opinions.	0	1	2	3	4
I negotiate with other people to reach an agreement.	0	1	2	3	4
I calmly tell others when I disagree.	0	1	2	3	4

**Table 2:** Health-Related Resiliency Scale in Spanish. INSTRUCCIONES: Lee cada frase cuidadosamente. Circula la respuesta que mejor indique la forma en la que has pensado y te has sentido (Nunca, Casi nunca, Raras veces, A veces, Siempre) durante los últimos 7 días. Recuerda que no hay respuestas correctas ni incorrectas.

Dimensiones	Nunca	Casi nunca	Raras veces	A veces	Siempre
**Dimensión de Sentido de Dominio**					
Visualizo mi futuro con esperanza.	0	1	2	3	4
Trato de ver el lado bueno de las cosas.	0	1	2	3	4
Puedo identificar los beneficios que resultan de mi situación.	0	1	2	3	4
Confió en mi capacidad para tomar decisiones.	0	1	2	3	4
Creo soluciones efectivas a mis problemas.	0	1	2	3	4
Derivo satisfacción de mis logros.	0	1	2	3	4
Logro lo que me propongo aún bajo estrés.	0	1	2	3	4
Pido ayuda cuando la necesito.	0	1	2	3	4
Me ajusto a lo me presenta la vida.	0	1	2	3	4
**Dimensión de Reactividad Emocional**					
Me mantengo tranquila aunque esté bajo estrés.	0	1	2	3	4
Manejo mis compromisos emocionales positivamente, a pesar del estrés.	0	1	2	3	4
Mantengo la calma durante situaciones difíciles.	0	1	2	3	4
Me recupero de las situaciones estresantes en corto tiempo.	0	1	2	3	4
Aun cuando enfrento dificultades, logro recuperar mi tranquilidad.	0	1	2	3	4
Manejo mis emociones durante situaciones difíciles.	0	1	2	3	4
Aun cuando enfrento dificultades me mantengo tranquila.	0	1	2	3	4
Mantengo mis emociones en control la mayor parte del tiempo.	0	1	2	3	4
Regulo mis emociones a tono con las situaciones que enfrento.	0	1	2	3	4
**Dimensión de Sentido Relacional**					
Confío en otras personas.	0	1	2	3	4
Puedo dejar que otros conozcan cómo me siento.	0	1	2	3	4
Puedo delegar en otras personas.	0	1	2	3	4
Los demás entienden mis preocupaciones.	0	1	2	3	4
Recurro a otros para consejos o asesoramiento.	0	1	2	3	4
Hay gente que me quiere y me cuida.	0	1	2	3	4
Mantengo relaciones duraderas.	0	1	2	3	4
Las relaciones con otras personas me ayudan a ver las cosas desde otro punto de vista.	0	1	2	3	4
Me siento cómoda en mis relaciones.	0	1	2	3	4
Respeto las diferencias en opiniones.	0	1	2	3	4
Negocio con otras personas para llegar a acuerdos.	0	1	2	3	4
Calmadamente puedo decirles a otros que estoy en desacuerdo.	0	1	2	3	4

### Procedure

The present sample was nested within the Hispanic-Latino longitudinal cohort of women (HLLC), that is part of the NeuroAIDS Research Program at the University of Puerto Rico (UPR), Medical Sciences Campus (MSC) with internal review (IRB) written approval Universidad de Puerto Rico, Recinto de Ciencias Medicas, Comite de Derechos Humanos (IRB), with all participants signing and receiving copies of stamped approved consent forms. Access to data from this cohort and related studies is managed by Dr. Valerie Wojna (valerie.wojna1@upr.edu). This unique cohort of Puerto Rican, Spanish-speaking, and HIV+ women was characterized with respect to the viral immune profiles, neurological function, and neuropsychological performance of its member women [2, 50]. Our cross-sectional data were collected between August 2010 and January 2011. All the participating seropositive women were screened at their primary HIV clinics at the Puerto Rico Medical Center (the Clinic for Sexually Transmitted Diseases and the Longitudinal Mother–Infant Clinic) and community-based organizations.

HIV+ women aged 21 to 65 years were recruited if they fulfilled the inclusion criteria of (1) presenting a nadir CD4 cell count of 500 cells/mm^3^ or less or a viral load of greater than 1,000 copies/ml while being active recipients of combined antiretroviral treatment (cART) (2) and having completed at least ninth grade (the minimum educational level required for some of the neuropsychological [NP] tests); active self-reported substance users were screened out. Women with a history of neurodegenerative disease or prior CNS infection (e.g. toxoplasmosis), neuropsychiatric disorders, active infections, or either current or past head trauma were excluded [2]. During her only site visit, each participant completed a demographic survey, the HRRS, and the Beck Depression Inventory I, Spanish version (BDI-I S) [48, 49].

### Study sample

All subsequent analyses are based on 45 consecutively recruited, HIV+ women from the HLLC with a mean age of 46 years (range, 32–63); described previously elsewhere [2]. Most of the participants were born and educated in Puerto Rico, went through the same or a similar education system, and had the same or a similar socio-economic status. Only 13% (n = 6) of the participating HIV+ women had been prescribed medication for symptoms of depression. The inclusion criteria screened out self-reported illegal substance users. However, drug testing revealed that a total of 16% (n = 7) of the HIV+ women were positive for one or more illegal substances on the day of the test. Drug testing indicated that five participants were positive for cocaine and two were positive for marijuana. The seven HIV+ women who tested positive for illegal substance use were included in this study after neuropsychological examination suggested that they were qualified to participate at the time of testing.

### Data analyses

All analyses were performed using Microsoft Excel (2010) and the Statistical Package for the Social Sciences (SPSS) software (version 17.0).

### Reliability

Reliability was calculated using Cronbach’s alpha [51] for the global score of the HRRS, each of the three-dimensional scores, and each of the 10 subscales.

### Content validity

Content validity was evaluated using Lawshe’s technique, which requires the objective judgment of subject matter experts (SME) to determine the congruence of items with conceptual definitions [52]. For the HRRS, SME were considered qualified to assess the HRRS based on their experiences working with HIV+ populations, as well as past work on resilience and psychometrics. The SME were instructed to pair individual items with dimensions/subscales that represented the measured construct by classifying items as essential or not essential. A content validity ratio was calculated from the responses provided by the SME.

### Predictive validity

Predictive validity was evaluated based on the correlation between the HRRS, the three dimensions of the HRRS, and the Beck Depression Inventory I, Spanish version (BDI-I S; [48, 49]), which is a widely used, and an established screening test that assesses the severity of the symptoms of depression in the 14 days prior to screening. We hypothesized that higher levels of resilience would be associated with lower levels of depression.

### Ethics

This study conformed to guidelines set forth in the latest version of the Declaration of Helsinki. The study design was approved by the University of Puerto Rico, Medical Sciences Campus, institutional review board (IRB). Participants provided informed consent to participate in the study after receiving a full explanation of all the procedures.

## Results

### Reliability

The results demonstrated excellent overall internal consistency for the total HRRS score (α = 0.95). Each of the dimensional scores also evidenced acceptable internal consistency (α ≥ 0.88). The internal consistency for each of the ten subscales was variable, ranging from acceptable to unacceptable. See Table 3 for details, including descriptive statistics for HRRS total score, each of the dimensions and subscales, and reliability statistics.

**Table 3:** Reliability and descriptive statistics.

Dimension/ Subscale	#	Reliability	Mean	SD	Max	Min
Total Score	30	0.95	97.16	15.33	120	53
Sense of Mastery Dimension	9	0.88	31.49	4.43	37	16
Optimism	3	0.78	10.69	1.44	12	7
Self-efficacy	3	0.78	10.64	1.72	12	5
Adaptation	3	0.69	9.98	1.80	12	4
Sense of Relatedness Dimension	12	0.88	36.71	7.44	48	9
Trust	3	0.85	7.87	2.73	12	0
Support	3	0.74	9.04	2.24	12	3
Comfort	3	0.67	9.82	2.07	12	4
Tolerance	3	0.82	10.20	2.14	12	0
Emotional Reactivity Dimension	9	0.93	28.51	6.47	36	11
Sensitivity	3	0.79	9.31	2.19	12	3
Recovery	3	0.81	9.87	2.05	12	4
Balance	3	0.86	10.09	3.34	27	3

SD: standard deviation; Max: maximum value; Min: minimum value; #: number of items

### Content validity

Data for content validity were collected from a total of 20 SME. Based on the Lawshe technique, when 20 judges have evaluated the instrument, the expected validity index is 0.42 [52]. An item analysis of the initial 50 items resulted in the elimination of three items from each dimension. An additional item was excluded so that there would be an equal number of items per subscale, resulting in three items per subscale, for a total of 30 items. All the dimensional and subscale content validity indices were above the 0.42 cut-off; see Table 4 for details.

**Table 4:** Content validity indices.

Dimension	Content Validity Index	Subscale	Content Validity Index
I. Sense of Mastery Dimension	0.81	1. Optimism	0.89
		2. Self-efficacy	0.88
		3. Adaptation	0.76
II. Sense of Relatedness Dimension	0.88	1. Trust	0.91
		2. Support	0.94
		3. Comfort	0.79
		4.Tolerance for differences	0.92
III. Emotional Reactivity Dimension	0.87	1. Sensitivity	0.89
		2. Recovery	0.82
		3. Balance	0.88
Total Resilience Scale	0.62		

### Predictive validity

Analysis revealed a significant negative correlation between HRRS total score and BDI-I-S (*r*(45) = -0.453, *p* < 0.003). All three dimension of the HRRS were also negatively correlated with the BDI-I-S (SDM: r(40) = -0.344, *p* < 0.03; SRD: r(40) = -0.390, *p* < 0.02; and ERD: r(40) = -0.428, *p* < 0.006).

### HRRS interpretation

The psychometric properties support the use of individual dimensions and the total scores of the HRRS. Higher scores are indicative of a greater level of a given dimension as well as the construct of health-related resilience.

## Discussion

Albeit preliminary in nature, the present study provides support for the HRRS as a measure to assess resilience among individuals living with chronic medical conditions. This measure is the first of its kind, having high levels of internal consistency as well as data to support its validity. Our group of SME examined and confirmed the HRRS’s content validity. The predictive validity was supported by correlations of the HRRS’s correlations in the expected direction with an established measure of depression. The measure is easy to interpret, with higher scores indicating greater resilience. The strengths of the study include the availability of the measure in two languages (English and Spanish) and founding preliminary psychometric properties of the measure based on a high-risk and vulnerable patient population in HIV+ Latina women from Puerto Rico. Minority populations, especially non-English speaking ones, are understudied across medicine [53–56], and when efforts are made to include these patient groups, measurement is rarely tailored to their unique cultural and linguistic experiences. The HRRS is a measure that addresses these notable voids in the medical literature.

Findings from this study highlight the inverse relationship between HRRS and depressive symptoms. This relationship is consistent with the limited existing literature. Lower levels of resilience have been noted in individuals receiving psychiatric care compared to healthy individuals [57]. Thus, an effort to improve health-related resiliency may provide an additional target for interventions aimed at addressing the needs of individuals with chronic health conditions. Early efforts have found that interventions that foster resilience have a positive impact not only on resilience but also on social support, as well as improving the mental health status of HIV+ individuals, and these treatment gains were shown to be sustainable for up to 3 months post-intervention [58]. Interventions that foster the development of health-related resiliency may represent a less threatening form of treatment, relative to more traditional psychiatric interventions, and may be easier to disseminate through health promotion efforts, especially in the clinical course of a given chronic medical condition. These efforts may be especially relevant to an already stigmatized patient population [59, 60–62], such as that consisting of those living with HIV.

Although this study has a number of strengths, its limitations must be acknowledged. The small sample size of this study represents a major limitation, and as such, all conclusions should be taken as being preliminary. Future studies should further assess the psychometric properties in larger, more diverse patient populations, including those consisting of individuals with chronic medical conditions other than HIV. Additionally, future research should address standardizing the HRRS in both languages (Spanish and English) and for both genders. Finally, given the one-time administration of the HRRS, findings from this study are unable to address the issue of the temporal stability of the measure. Some conceptualize resilience as a personality trait [43, 45, 61], implying that there is an expected consistency in the pattern of resilience over time. When specifically examining health-related resilience, however, one might expect resilience to vary as a function of the chronicity of a given disease state as well as with respect to the expected fluctuations in health status common to many chronic medical conditions, including HIV. As such, resilience may vary with time across dimensions or in terms of a specific dimension [62]. Future studies should longitudinally assess health-related resilience, especially in the context of disease progression.

## Conclusion

Limitations notwithstanding, the HRRS is the only bilingual (Spanish and English) measure to assess health-related resiliency in adults. The study described in this manuscript lends credence to the pertinence of the preliminary psychometric properties as determined by the instrument, which is itself intended as a measure to be used with HIV+ Puerto Rican women, an underserved and understudied population. Despite the homogenous patient population upon which the psychometric properties are based, the measure is likely to be of relevance across diverse chronic medical conditions. Health-related resiliency may be a less-threatening and less-stigmatizing target for interventions aimed at improving the wellbeing and quality of life of individuals living with chronic medical conditions.
